# Conceptualising the Impact of Arousal and Affective State on Training Outcomes of Operant Conditioning

**DOI:** 10.3390/ani3020300

**Published:** 2013-04-11

**Authors:** Melissa J. Starling, Nicholas Branson, Denis Cody, Paul D. McGreevy

**Affiliations:** 1 Faculty of Veterinary Science, University of Sydney, Sydney NSW 2006, Australia; E-Mail: paul.mcgreevy@sydney.edu.au; 2 Deakin Research, Deakin University, Geelong VIC 3217, Australia; E-Mail: Nick.Branson@deakin.edu.au; 3 Indice Ecotech Pty Ltd., East Richmond VIC 3121, Australia; E-Mail: denis.cody@internode.on.net

**Keywords:** arousal, affective state, operant conditioning, animal training, dogs, horses

## Abstract

**Simple Summary:**

This article discusses the impacts of arousal and emotional state on training animals using methods based on reward and punishment. Three-dimensional graphs are provided to offer a visual means to illustrate how arousal and emotional state may influence the effectiveness of reward and punishment depending on the behaviour being trained. Dogs and horses are used to illustrate this with reference to commonly trained behaviours in a predatory and a prey animal.

**Abstract:**

Animal training relies heavily on an understanding of species-specific behaviour as it integrates with operant conditioning principles. Following on from recent studies showing that affective states and arousal levels may correlate with behavioural outcomes, we explore the contribution of both affective state and arousal in behavioural responses to operant conditioning. This paper provides a framework for assessing how affective state and arousal may influence the efficacy of operant training methods. It provides a series of three-dimensional conceptual graphs as exemplars to describing putative influences of both affective state and arousal on the likelihood of dogs and horses performing commonly desired behaviours. These graphs are referred to as response landscapes, and they highlight the flexibility available for improving training efficacy and the likely need for different approaches to suit animals in different affective states and at various levels of arousal. Knowledge gaps are discussed and suggestions made for bridging them.

## 1. Introduction

Trained animals are important contributors to work, sport and recreation. As a leading example, domestic dogs (*Canis familiaris*) have a long association with humans that depends largely on our ability to train them. This training ranges from conditioning simple behaviours that optimise sharing a living space with them to highly specific and complex behavioural sequences that capitalise on the abilities of dogs that we lack, such as speed, agility and a keen sense of smell.

Current approaches for training animals are generally anchored in operant conditioning [1]. The science behind operant conditioning, while detailed, sound, and very useful, is not a complete model, missing biological and psychological principles beyond the behavioural principles developed that may help further our understanding of the origins of behaviour [2, Chapter 1]. For example, in dogs, it may fail to fully explain why one dog may relate more to one trainer than another, despite both trainers using the same operant techniques [3]. It has long been held that behavioural output is also a product of affect, which comprises two components, arousal and emotional valence [4,5]. Unlike operant conditioning outcomes, arousal and emotional valence are more challenging to quantify. Arousal refers to physiological and psychological activation into a state of general wakefulness or attention [6]. In non-human animals, it is generally measured by physiological signs known to increase in association with arousal, such as tachycardia, hypotension and pupil dilation [7,8] and changes in skin-conductance levels [9].

Arousal was first conceptualised as a generalised construct, in which a single dimension accounts for arousal in all circumstances. The Yerkes-Dodson Law is the most widely recognised general arousal construct [10]. The arousal level associated with the highest performance on a task is considered the optimal arousal level for that task [11]. Where arousal is below the optimal level, under-stimulation may result in slow performance or lack of interest in performing at all. Where arousal is above the optimal level, performance suffers due to narrowing of focus so that only a few cues can be attended to (see [12] for review and discussion). Optimal arousal levels are considered to be task-specific, such that more challenging tasks are performed more successfully at low arousal levels and simpler tasks can be performed successfully at higher arousal levels. This has been described as the inverted-U relationship [13].

Emotional valence captures the notion of positive and negative affective states. Intuitively, one might predict these to have an immediate impact on an animal’s likelihood of responding to learned stimuli. However, it has only recently been possible to consider the contribution of emotional valence to training outcomes as advances in the area of animal cognition have provided possible indicators of both positive and negative affective states. The most promising of these indicators may be cognitive bias, which refers to the tendency for affective state to influence cognitive processes [14]. Recent animal studies have found that one class of cognitive biases, known as judgment or expectation bias, can be measured objectively in animals [15,16,17,18,19,20,21,22,23,24,25]. A negative affective state is associated with more negative outcomes expected, and positive affective states with more positive outcomes expected, e.g., [19,21].

Cognitive biases are expressed in response to both short-term changes in an individual’s level of anxiety (state anxiety) and long-term differences in an individual’s tendency to experience anxiety (trait anxiety) [26]. Although both forms of anxiety are fundamentally different, their effects on learning processes have been shown to be the same (e.g., [27]). There is evidence suggesting that judgment bias in animals correlates with affective state. For example, pessimism has been found to be higher in dogs that also score highly in measures of separation-related distress [18], and starlings prone to stereotypic behaviour are more pessimistic than their non-stereotyping conspecifics [20]. The findings of Mendl *et al*. [18] show how affective state may be correlated with behavioural output, which not only has implications for how we assess the welfare impacts of anxiety-related behaviour in dogs, but also how a dog’s affective state may relate to the behaviour it displays both in training scenarios and in everyday life.

The results of these studies highlight the importance of establishing a predictive model of animal behaviour that includes the influences of arousal and affective state and their potential interactions with each other. At the time of writing, a few models provide starting points. Mendl *et al*. [28] proposed a model that integrated discrete emotions and dimensional states of “core affect”, to encompass both emotional valence and arousal. Combining arousal and valence to give core affect was proposed as a kind of currency that enables animals to prioritise actions based on discrete emotions in response to current events. Furthermore, the frequency of an animal experiencing various discrete emotions may influence its background mood, thus giving rise to emotional states unattached to particular events, which in turn influence the discrete emotions experienced.

An arousal construct has been proposed in which there are multiple arousal types, each for a specific type of response (e.g., feeding, locomotion, flight response *etc.*), under the influence of a general arousal system (see [29] for review). Combining this arousal construct with the contribution of the affective state on cognitive processes in animals presents an opportunity to form a comprehensive picture of the effect of these variables on training outcomes. An affective neuroscience construct developed by Panksepp [2] uses the concept of modes based on neural substrates to classify specific emotional states related to common behaviour in mammals. This may be viewed as a dimension additional to the multiple arousal constructs based also on specific responses. This is potentially a helpful start in integrating emotional states and arousal with behavioural output. However, in a practical sense, it is difficult to apply either construct to animal training scenarios. We may align the emotional mode of SEEKING with the arousal associated with foraging, for example, and consider it a harmonious state for training to occur, where trainers may get maximal commitment and focus from their animal trainees. Beyond such broad adoptions, there are difficulties associated with specifics, such as how to identify when the animals are in such a harmonious state and when they have slipped out of it, to where, and why. It is likely there is overlap between neural substrates and associated goal-oriented behaviour. For example, there is likely to be an overlap between RAGE and FEAR systems in defensive behaviour and RAGE and SEEKING systems in inter-male aggression [2]. These overlapping modes, while accepted as the nature of emotional states, may serve to confuse practitioners in application. 

In the current article, we attempt to build on these foundations by offering specific examples that are regularly encountered by animal trainers. Including a multiple arousal type or multiple emotional substrate construct is beyond the scope of this paper. Rather, we hope to offer an intermediary framework for integrating constructs of arousal and emotion with operant conditioning in a context familiar to animal trainers in order to encourage adoption of more inclusive paradigms than operant and classical conditioning alone.

The interpretation of operant conditioning terms can be ambiguous. Traditionally, reinforcers and punishers in operant conditioning have been considered strictly as stimuli, thus avoiding the difficulties with defining motivation and affective states [30]. However, it has been shown, for example, that negative reinforcement (defined as increasing the frequency of a behaviour by withdrawing a stimulus) is associated with the onset of safety, which could be considered positive reinforcement [30]. It is highly likely that there are emotional components to operant conditioning, and that affective states themselves can act as reinforcers or punishers. This has been considered in research into human drug addiction, where taking drugs assuages negative affect created by psychological conditions (e.g., [31]).

In the current article, we consider the influences of arousal and affective state on the processing of reinforcement and punishment. Here we take the simplest view of classifying reinforcement and punishment as positive (presented) or negative (withdrawn). Thus trainers often speak of the four quadrants of operant conditioning: positive reinforcement, negative reinforcement, positive punishment, negative punishment [1]. The current article does not consider the effects of varying sensitivity to reinforcement and punishment between individuals. Evidence suggests baseline sensitivity to rewards may affect personality and individual tendencies towards broad behaviours such as reward-seeking, novelty-seeking and impulsivity (e.g., [32,33,34]). This may further affect individual animals’ responses to different applications of operant conditioning, reflecting differences in how signals are assessed and the likelihood of approach-versus-avoidance behaviours. This is considered beyond the scope of the framework presented here.

This paper presents a conceptual model of how, depending on the training methods used, affective state and general arousal may influence training outcomes, as judged by the probability of the animal displaying a desired behaviour on cue. The model follows on from earlier work that provided conceptual, three-dimensional graphics using four quadrants to chart a horse’s responsiveness to various cues from two reins and the rider’s legs and seat [35], and builds on broader, integrative constructs including arousal and affective state [2,28]. For the purposes of presenting a simplified concept, “desired behaviours” in this case are a series of target behaviours that represent a variety of responses commonly required of dogs or horses, the two species that are arguably the most commonly trained. “Undesired behaviour” is any behaviour that significantly detracts from or is incompatible with the target behaviour. The arousal-producing stimulus is assumed to be general in nature, for example, a large number of nearby competing stimuli (such as other animals and people undertaking energy-intense activities). 

## 2. Experimental Section

A series of response landscapes similar to the response surfaces used by Nijhout [36] and discussed by Overall [37] was created using the program Mathematica 8 (Wolfram Research, Champaign, IL, USA) to represent how affective state and arousal levels may affect the efficacy of each operant-conditioning quadrant in training animals to perform particular behaviours. In this case, efficacy is considered the probability of the animal performing the desired behaviour, assuming the trainer is adept at applying the method in question in the sort of uncontrolled (non-laboratory) environments in which animal training often takes place. It was assumed that competing stimuli, such as other animals and handlers and smells and sounds of ethological importance to the trainee animals, would be present and may play a role in increasing arousal or inducing changes in affective state. Dogs and horses were used as models to capture commonly encountered species-specific responses as well as commonly trained behaviours.

The graphs were based first on two matrices of putative data assembled by the authors: one deals with the probability of the dog performing the desired behaviour given different levels of arousal for each of the four quadrants (positive reinforcement, negative reinforcement, positive punishment, negative punishment), and the other deals with the probability of the desired behaviour given different levels of affect for each operant-conditioning quadrant. The data for both arousal and affective state were represented on a hypothetical 10-point scale, with 0 being the lowest arousal level and most negative affective state and 10 being the highest arousal level and most positive affective state. 

Combining these two-dimensional matrices into three-dimensional matrices was achieved by first supplying a skeleton dataset of the probability of the desired behaviour for each quadrant (given hypothetical arousal levels of 0–10) and the probability of the desired behaviour being performed for each quadrant (given each point on the hypothetical affective state scale of 0–10), as discussed previously. Thus, each quadrant had an associated dataset of probability, given affective state, and probability, given arousal level. These values were based on discussions with trainers and observations of trialing dogs and experiences with horse riding, as well as drawing from the equitation science and dog training literature. The dataset was then filled out by generating numbers to fit the distributions defined in the two-dimensional matrices using a mathematical function with a semi-Bayesian statistic. It was assumed that affective state and arousal are independent, or at least have very low co-variance. This has not been shown empirically, but can be later tested with the collection of relevant data. Using this assumption, it follows that the probability of a behaviour occurring due to arousal A and affective state B equals the probability of a behaviour occurring due to arousal state A multiplied by the probability of a behaviour occurring due to arousal state B. This follows the form of the Law of Multiplication in probability, which states that for independent events, the probability of Event A is not affected by the occurrence of Event B, so P(A and B) = P(A) × P(B). This was used for all target behaviours.

### Target Behaviours

The target behaviours chosen to map response landscapes were selected to represent a diversity of the types of behaviours animals are frequently trained to perform, and to capture behaviours that lend themselves more to different training methods. Given the earlier discussion of task complexity in the Yerkes-Dodson law, target behaviours were chosen to represent different complexities. For example, tracking, while being ostensibly a simple matter of following a scent trail, is considered to require a high level of attention to a narrow set of stimuli in comparison to the many, diverse, and at times distracting stimuli concurrently present in a tracking environment. Such distractions include other social objects, potential threats and opportunities for reinforcement unrelated to the target behaviour. In contrast, targeting is a fast and simple behaviour that is unlikely to require exclusive attention. Reinforcers and punishers considered as possible tools in the training of each behaviour were restricted to those tools most readily available. As such, stimuli such as electronic collars were not considered, because of their cost or restricted availability. Consideration was given to the availability of reinforcers and punishers to trainers depending on the target behaviour and readily available training tools. A description of the target behaviours and the reinforcers and punishments considered available for each operant training category are shown in Table 1.

**Table 1:** Summary of the conditions considered in the formation of the response landscapes.

Species (Figure number)	Behaviour	Description	PR	NR	PP	NP
Dog (1,2)	Heel on leash	Dog to walk on loose leash in heel position	Food, play, or access to toy, praise and affection, release to engage in natural behaviours	Social pressure, physical pressure from leash	Loud noise, physical correction—leash or otherwise, shout	Withdrawal of access to reinforcers mentioned in PR
Dog (3)	Heel off leash	Dog to walk in heel position unaided	Food, play or access to toy, praise and affection, release to engage in natural behaviours	Social pressure	Loud noise, shout, physical correction	Withdrawal of access to reinforcers mentioned in PR
Dog (4)	Tracking	Dog to follow scent trail to source	Food, play or access to toy, praise and affection, release to engage in preferred behaviours	Social pressure, separation from handler	Loud noise, shout, physical correction	Withdrawal of access to reinforcers mentioned in PR
Dog (5)	Stay	Dog to remain in stationary position	Food, play or access to toy, release from stay, praise and affection	Social pressure, physical pressure from leash	Loud noise, physical correction—leash or otherwise, shout	Withdrawal of access to reinforcers mentioned in PR
Horse (6a)	Target training (in-hand)	Horse to touch target with its nose	Food, scratching of the withers and neck	Pressure from the headcollar or bridle	Physical correction	Withdrawal of access to reinforcers mentioned in PR
Horse (6b)	Walk forward (under-saddle)	Horse to walk forward in response to pressure from the rider’s legs	Food, scratching of the withers and neck	Pressure from rider’s legs	Physical correction	Withdrawal of access to reinforcers mentioned in PR

Each target behaviour is named and described and the forms of positive reinforcement (PR), negative reinforcement (NR), positive punishment (PP) and negative punishment (NP) considered common and easily accessible are listed.

## 3. Results

The response landscape graphs generated from the original two-dimensional matrices were considered by the authors to represent a possible model illustrating the effects of arousal and affective state on the efficacy of different operant-training approaches in dogs and horses. The graphs show arousal levels on the x-axis, affective state on the z-axis, and the probability of the animal performing the desired behaviour on the y-axis. The resulting landscape shows how the probability of the animal performing the desired behaviour follows knolls, peaks and valleys, depending on the arousal and affective states of the animal and the operant-conditioning technique being used to train a specific behaviour. Response landscape graphs are shown in the text, but may be accessed in interactive form at the following URL: http://hdl.handle.net/2123/8989.

**Figure 1:**
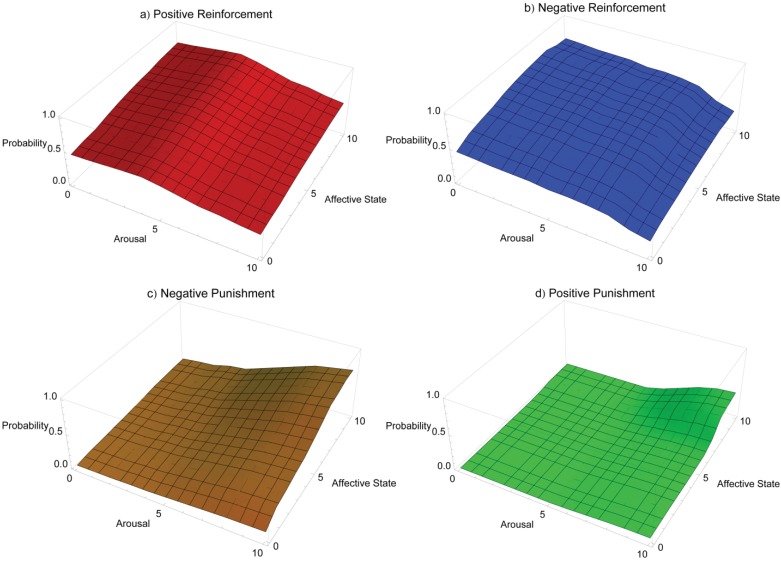
A breakdown of the conceptual response landscape for training a dog to heel on leash, showing each operant training quadrant on a separate graph.

Figure 1 shows a breakdown of the response landscape for training heeling on leash in dogs, displaying individual response landscapes for each operant training approach. In the figure, the y-axis tracks the possible probability of a dog heeling on leash depending on the dog’s affective (z-axis) and arousal states (x-axis), both of which are shown on a simple, representative scale of 0–10, where 0 is low arousal and a very negative affective state and 10 is high arousal and a very positive affective state, respectively. Figure 1(a) shows the positive reinforcement response landscape, characterised by high probabilities of the dog heeling on leash, peaking at moderate arousal where arousal matches the required activity level, and positive affective state where the dog may be most attentive to opportunities to access reinforcers. Figure 1(b) shows the negative reinforcement landscape, which steadily decreases in efficacy as arousal and affective state values increase. Increased arousal may result in a higher likelihood of behaviours more active than heeling on leash, and more positive affective state may be associated with greater distractibility as the dog attends to stimuli in the environment that may signal access to environmental reinforcers. These conditions may combine to reduce the dog’s attention to negative reinforcement. Figure 1(c) shows the response landscape of negative punishment, which is most effective at high arousal and very positive affective state. In this condition the dog is likely to be attentive to opportunities to access reinforcement, yet may be prone to extraneous behaviour related to an arousal state higher than is appropriate for on-leash heeling. Negative punishment may aid in reducing undesired behaviour while maintaining desired behaviour. Efficacy may decrease with decreased values for arousal and affective state as dogs become more sensitive to reinforcement loss as their affective state declines, and less likely to persist in activities as arousal decreases. Figure 1(d) shows the response landscape for positive punishment. Efficacy is very low where affective state is negative and arousal is low as the dog is less likely to display any behaviours and more likely to be sensitive to punishment. Efficacy increases only at high arousal and very positive affective state where the dog may be more likely to display excessive undesired behaviour that may benefit from strategic suppression. Response landscape graphs may be accessed in interactive form at the following URL: http://hdl.handle.net/2123/8989.

**Figure 2:**
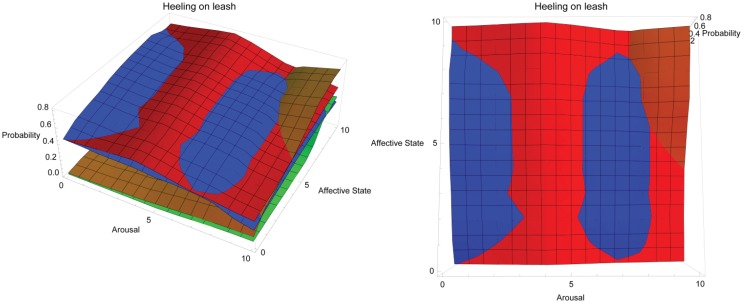
Combined conceptual response landscape for training heeling on leash in dogs using different operant training methods.

Figure 2 shows the conceptual response landscape for training heeling on leash in dogs with all operant conditioning approaches combined into the one landscape. This illustrates how the shapes of each operant conditioning response landscape may interact with one another, showing where approaches may be most effective compared to other approaches. In the figure, two views of the same response landscape are shown: aerial view on left and side view on right. Red = positive reinforcement, blue = negative reinforcement, orange = negative punishment, green = positive punishment. The y-axis tracks the probability of a dog heeling on leash depending on the dog’s affective (z-axis) and arousal states (x-axis), both of which are shown on a simple, representative scale of 0–10, where 0 is low arousal and a very negative affective state and 10 is high arousal and a very positive affective state, respectively. This behaviour is performed in the presence of a leash, which may provide an effective means of applying negative reinforcement. Both positive and negative reinforcement are expected to gradually decrease in efficacy as arousal increases and affective state becomes positive, resulting in dogs displaying more energetically costly behaviour that may be at odds with steady, controlled movement, but positive reinforcement is predicted to peak at moderate arousal rather than low arousal. This contrasts with the response landscape for negative reinforcement and accounts for the apparent division in the negative reinforcement landscape by a knoll that erupts through the positive reinforcement landscape. Response landscape graphs may be accessed in interactive form at the following URL: http://hdl.handle.net/2123/8989.

**Figure 3:**
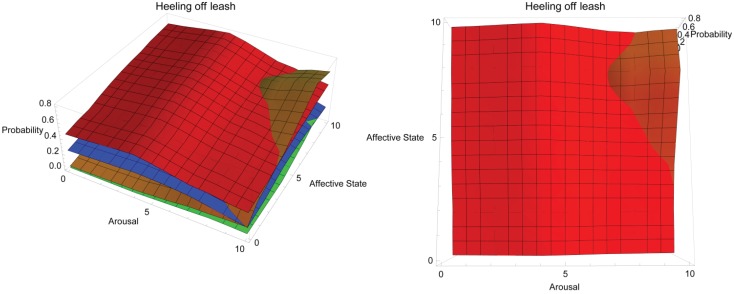
Conceptual response landscape for training heeling off leash in domestic dogs using different operant training methods.

Figure 3 shows the conceptual response landscape for training a dog to heel off leash. In the figure, two views of the same response landscape are shown: aerial view on left and side view on right. Red = positive reinforcement, blue = negative reinforcement, orange = negative punishment, green = positive punishment. The y-axis tracks the probability of a dog heeling off leash depending on the dog’s affective (z-axis) and arousal states (x-axis), both of which are shown on a simple, representative scale of 0–10, where 0 is low arousal and a very negative affective state and 10 is high arousal and a very positive affective state, respectively. This behaviour requires the dog to willingly approach and remain in close proximity to the handler, as the behaviour by definition must be performed without the aid of restraining tools. Positive reinforcement dominates the response landscape in efficacy as it is well suited to encouraging approach behaviour. Its efficacy peaks at moderate arousal and positive affective state. At high arousal and very positive affective state, negative punishment may prove effective as it offers a means to decrease extraneous behaviour that may result from increased activity directed towards seeking and acquiring reinforcers. Restricting access to environmental reinforcers may be more difficult to achieve with the dog off leash, but may remain effective where it is possible. Response landscape graphs may be accessed in interactive form at the following URL: http://hdl.handle.net/2123/8989.

**Figure 4:**
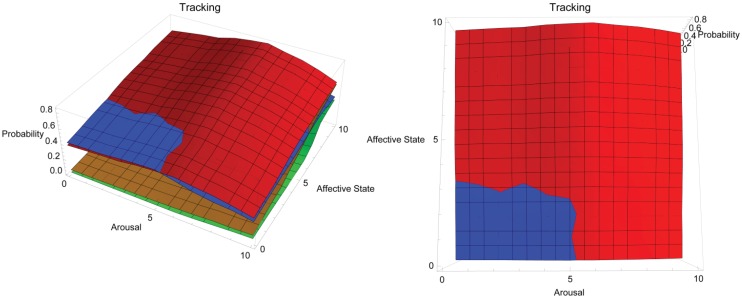
Conceptual response landscape for training a dog to track using different operant training methods.

Figure 4 shows the conceptual response landscape for training a dog to track a target scent through the environment. Like heeling off leash, this behaviour may be difficult to train with the use of physical training aids. In the figure, two views of the same response landscape are shown: aerial view on left and side view on right. Red = positive reinforcement, blue = negative reinforcement, orange = negative punishment, green = positive punishment. The y-axis plots the probability of a dog successfully tracking depending on the dog’s affective (z-axis) and arousal states (x-axis), both of which are shown on a simple, representative scale of 0–10, where 0 is low arousal and a very negative affective state and 10 is high arousal and a very positive affective state, respectively. This activity requires extended focus from the dog, which may be most efficiently supported across most states by positive reinforcement, as this is likely to encourage the dog to persist in the behaviour even when reinforcement is intermittent. It is possible that with low arousal and affective state values, negative reinforcement may be effective in creating the necessary motivation for the dog to perform this behaviour. At high arousal and affective state values, negative punishment in the form of taking the dog away from potential reinforcement may be effective in suppressing undesired behaviour related to inappropriately high arousal and positive affective state combining to distract the dog from the task. Response landscape graphs may be accessed in interactive form at the following URL: http://hdl.handle.net/2123/8989.

**Figure 5:**
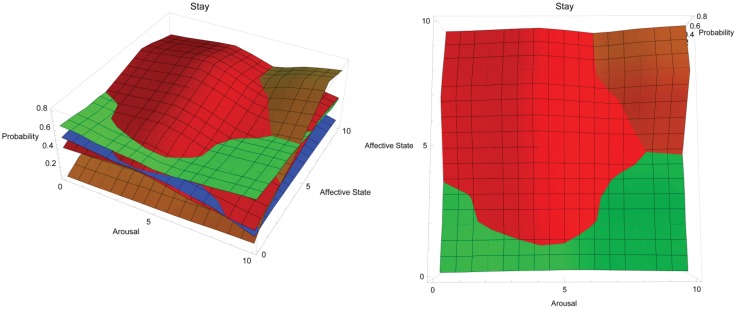
Conceptual response landscape for training dogs to stay (remain stationary) using different operant training methods.

Figure 5 shows the conceptual response landscape for training a dog to stay. In the figure, two views of the same response landscape are shown: aerial view on left and side view on right. Red = positive reinforcement, blue = negative reinforcement, orange = negative punishment, green = positive punishment. The y-axis tracks the probability of a dog staying in place depending on the dog’s affective (z-axis) and arousal states (x-axis), both of which are shown on a simple, representative scale of 0–10, where 0 is low arousal and a very negative affective state and 10 is high arousal and a very positive affective state, respectively. Stay is a stationary behaviour, so theoretically it may be possible to train this behaviour by suppressing all behaviour with the use of positive punishment, particularly if the dog is in a negative affective state and low arousal and is therefore not compelled to move very much in the first place. Willing cooperation may be useful at higher arousal and more positive affect, but may be difficult to obtain at the extreme of this condition using positive reinforcement where active seeking of reinforcement may become more likely. Negative punishment may be very effective in these conditions by encouraging impulse control. Response landscape graphs may be accessed in interactive form at the following URL: http://hdl.handle.net/2123/8989.

**Figure 6:**
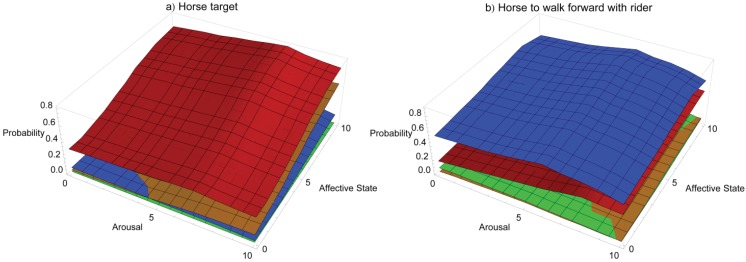
Conceptual response landscapes for training two common behaviours in the domestic horse using different operant training methods.

Figure 6 offers the contrast of two horse examples, training a horse to touch a target with its nose and training a horse to move forward with a rider in the saddle. In the figure, Red = positive reinforcement, blue = negative reinforcement, orange = negative punishment, green = positive punishment. The y-axis tracks the probability of the horse responding appropriately depending on its affective (z-axis) and arousal states (x-axis), both shown on a simple, representative scale of 0–10, where 0 is low arousal and a very negative affective state and 10 is high arousal and a very positive affective state, respectively. Figure 6(a) shows the training of a horse to touch a target on cue with its nose. Figure 6(b) shows the training of a horse to walk forward on cue from a rider in the saddle. Horses are generally more prone to reacting with flight than dogs, as prey animals are dependent on flight for safety. This is shown in the low efficacy of punishment-related training that may be likely to trigger evasive action. The targeting response landscape is dominated by positive reinforcement, as it is an approach behaviour and thus most suited to seeking reinforcement. In contrast, the response landscape in Figure 6(b) is dominated by negative reinforcement, as it is difficult to deliver any strong positive reinforcers from the saddle. Response landscape graphs may be accessed in interactive form at the following URL: http://hdl.handle.net/2123/8989.

## 4. Discussion

The response landscapes presented here are conceptual only, and are presented to provide a launching platform for further discussion and the collection of data to test the predictive powers of this model. The response landscape can be used to describe graphically how arousal and affective state may influence the efficacy of different operant conditioning training methods under typical environmental conditions. One important caveat is that all operant conditioning approaches are expected to be effective, and the effectiveness of one approach over others is likely to be linked to the previous conditioning of the animal, the skill of the handler in applying that approach, and how the handler routinely interacts with the animal (e.g., [38]). The response landscapes may also be adapted to describe how the above factors and others involved in training animals affect the efficacy of different methods and their graphical nature may offer an accessible way to discuss the finer points of training with professional trainers who may lack a strong scientific background.

The response landscapes shown here highlight how the ability to deliver reinforcement or punishment can influence the efficacy of training methods. It could be argued that this deviates from a strictly theoretical approach. However, the emphasis here is on the interplay of affective state, arousal and learning in contexts regularly encountered by animal trainers. Necessarily, this interplay includes the ability of the trainer to apply reinforcement and punishment and takes into account readily available training aids, as well as the variables introduced by a dynamic environment in which training often occurs. The response landscapes for heeling on leash (Figure 2) and heeling off leash (Figure 3) are similar, which is an artefact of the behaviours being very similar and trained in the same environment, but the omission of a leash changes the efficacy of negative reinforcement. This is highlighted again in Figure 6(b) where negative reinforcement dominates the response landscape for training a ridden horse to move forward. The reinforcements the rider has control of from this position are extremely limited (see Table 1). In contrast, the response landscape in Figure 6(a) for training a horse to target is dominated by positive reinforcement. The reinforcements available to the trainer in training this behaviour are much broader.

All response landscapes highlight that the efficacy of different operant training approaches may change with arousal levels and affective state, and these changes may differ, depending on the type of behaviour being trained. For example, a “knoll” can be identified in the response landscapes for positive and negative reinforcement while training responses that may run counter to those prompted by environmental stimuli. Examples shown here are heeling and remaining stationary (stays), where self-control may be required to perform steady locomotory responses or stationary behaviours in the presence of environmental stimuli that may trigger contrary movement and a higher associated level of arousal, such as fast-moving objects or other social objects. Self-control is thought to be a finite resource subject to depletion [39,40], reflecting additional complexity or difficulty to a task. This knoll may be a manifestation of the Yerkes-Dodson inverted-U, but with the added dimension of affective state. The inconsistency in this pattern across all training methods and behaviours may be explained by a more sophisticated arousal construct than a general one. The Yerkes-Dodson law is not universally accepted in the literature [12,41], and some researchers have argued that arousal is an adaptive process that has evolved to help animals solve problems that they regularly encounter, and therefore high arousal states produce the behaviour needed to cope with specific problems [12]. This has led to more modern arousal constructs that include both a general arousal construct associated with the central nervous system [42] and specific arousal types under the influence of the general arousal construct. To the authors’ knowledge, no such comprehensive arousal construct exists for animals in training, but the development of such a construct may aid in understanding the intricacies of how arousal and affective state influence training outcomes.

The response landscapes may be interpreted in two key ways. One way is as a guide to the possible efficacy of different training approaches, depending on the arousal and affective states of the animal during a training session. The second is as a map to where the animal’s arousal and affective state will best complement the use of a particular training method, for example, there are good reasons to preferentially use positive reinforcement. It is expected from research on cognitive bias in animals, that most animals in a negative affective state are more likely to interpret ambiguous signals as predictors of a negative outcome [18,21,24,43]. For example, many urban dogs encounter ambiguous signals on a day-to-day basis, such as the subtleties of body language in an unknown dog or human, unidentified sounds and smells, and visual stimuli such as a white paper bag on the ground that may or may not contain discarded food. It can be argued that training interactions with humans also contain an element of ambiguity for dogs, such as interpreting hand signals or verbal cues and reading body language [3]. As such, it is predicted that, in general, all operant training approaches will be negatively affected by a negative affective state. This has implications for the long-term effects of selecting training methods. 

It is noted that all quadrants in operant conditioning are effective in that, when exposed to salient stimuli, animals will learn regardless of the quadrant used and animals have evolved to respond to all quadrants. Quadrant-based training paradigms do not represent a best practice approach to humanely training new behaviours, maintaining learned behaviours, or suppressing unwanted behaviours. The response landscapes for at-liberty behaviours in particular highlight this by showing how efficacy may vary given the arousal level and affective state of the animal. It is not the intention of the authors to promote a particular quadrant over others, but rather to promote positive affective state and appropriate arousal levels for the desired behaviour to facilitate training by maximising the likelihood of the behaviour occurring but also to help the trainer in troubleshooting. A shift in training towards building behaviours and reliability from the ground up where arousal and affective state may be considered basic foundations and training approach in an operant conditioning framework may be considered a secondary system that may benefit both animals and their trainers. It may promote the ethical treatment of animals by encouraging trainers to put their animals’ emotional needs first and also help trainers to obtain the desired behaviours and associated reliability in performance by bringing animals to a place in the core affect landscape, where the animals are at their most responsive, first. We propose that trainers radiate out to other places in the core affect landscape later in training. This is not necessarily a different way of training, but the intent is to shift the focus away from first getting behaviours by whatever operant conditioning means necessary to first building the appropriate emotional and arousal foundations. Arguments that certain operant conditioning approaches are more effective than others may be true in some circumstances (e.g., [44]), yet may fail to take into account the merits of first manipulating arousal levels and affective state to create conditions in an animal that best complement training methods associated with ease of application and promotion of positive affective state and appropriate levels of arousal. This may be achieved through various means, for example, arousal levels can be manipulated by desensitising or using classical counter-conditioning to allow the dog to feel comfortable in a stimulating, outdoor environment so that the dog is not compelled to attend to competing stimuli. This may also improve affective state by reducing the number and/or intensity of threatening stimuli in the environment where training takes place.

Long-term effects of the operant training approaches are not considered in the current conceptual response landscapes. However, we predict that to some extent at least there are feedback mechanisms involved in both the long and short term. Regular exposure to positive reinforcement is likely to aid in maintaining a positive affective state, and regular exposure to aversive stimuli is likely to aid in maintaining a negative affective state, generating long-term positive and negative moods [2]. As such, aversive methods aimed at suppressing behaviour may be effective in the short-term, but repeated use may push animals into a general negative mood. There is evidence that punishment used in dog training is associated with increased incidence of aggression, particularly directed towards the handler [45,46,47,48], while compromising performance in obedience and protection work [48], and reducing willingness to approach strangers and engage in playful activities outside of training [45]. Furthermore, punishment-based training is believed to have the potential to create general anxiety in dogs [47] and has been associated with distress where timing is inexact [49,50]. Inappropriate use of aversive stimuli is of particular concern in horse training [51,52]. These indications suggest repeated and prevalent use of punishment in training may be associated with inducing a negative affective state in animals. It may also hamper the formation of the human-animal bond, which in turn is thought to play an integral role in trainee focus and, therefore, training outcomes [38]. In contrast, reward-based training has been shown to be associated with improved focus and ability to learn a new task [45,53]. The broaden-and-build theory in human affective neuroscience holds that positive emotional states encourage behavioural flexibility and resilience (see [54] for review). To the authors’ knowledge, this has not been studied directly in non-human animals, but some similarities may be identified. Positive affect appears to have benefits for focus and performance in learning new tasks. Play, which is strongly associated with a positive emotional state, may have several positive effects at the level of the individual animal. This may be particularly apparent in a social arena where animals that play may be more adept at acquiring mates, coping with intraspecific competition, and making affiliations, but effects may also be seen in a decrease in problem-solving ability and habituation to novelty and increase in fearfulness in social situations in animals that did not play (see [2] for review, pp. 294-295). Extant work in this domain focuses on rats, but is likely to be applicable to other mammals. Whether these effects are due to positive affect or the skills learnt in play is unclear, but perhaps the differentiation is irrelevant for inclusion in the broaden-and-build theory.

These feedback systems can explain some of the features in the response landscapes presented here. Where the animal is already in a negative affective state or it perceives competing stimuli as threatening, the animal is expected to pay more attention to threatening stimuli, as has been shown in human studies [27,55], and the resulting arousal would be geared towards readying to act in order to avoid or escape from potential danger. As arousal increases in the condition of negative affective state, affective state is likely to become more negative, and the probability of the animal offering a desired behaviour is likely to decrease across all operant-conditioning quadrants. That said, the effectiveness of negative reinforcement is postulated to increase as the need for the animal to escape threatening stimuli intensifies until arousal levels are high enough to provoke flight or fight responses.

## 5. Conclusions

Response landscapes may offer a framework both for discussing the effects of different factors on training efficiency and communicating those effects to laypeople. Recent studies suggest a need for a predictive model of behaviour that incorporates arousal, affective state, and operant conditioning. Response landscapes are used here as a graphical means to represent a preliminary, conceptual model incorporating these factors. Simple measures of affective state and arousal would provide the means to test the current predictions and enable the adoption of a more complete model of species-specific behaviour during training than that offered by operant conditioning alone. A comprehensive arousal construct for individual species, including multiple types of arousal as well as general arousal, may aid in understanding how animals will respond to various training methods in the presence of different competing environmental stimuli. 

## Supplementary Files

Supplementary File 1
